# Circulating tumor DNA as a tool for precision management in bladder cancer: A narrative review

**DOI:** 10.1177/17562872261493705

**Published:** 2026-10-07

**Authors:** Sri Saran Manivasagam, Fatemeh Sheikhsaran, Jay D. Raman, Alireza Aminsharifi

**Affiliations:** 1 Department of Urology, 12311 Penn State Health Milton S. Hershey Medical Center, Hershey, PA, USA; 2 Dorothy A Pauley Department of Urology, 6889 Virginia Commonwealth University, Richmond, VA, USA; 3 Cellular and Molecular Therapeutics Branch, NHLBI, 2511 National Institutes of Health, Bethesda, MD, USA

**Keywords:** circulating tumor DNA, bladder cancer, recurrence monitoring, targeted therapy, liquid biopsy, minimal residual disease

## Abstract

Circulating tumor DNA (ctDNA) has emerged as a potential paradigm-shifting biomarker for precision management of bladder cancer, particularly muscle-invasive bladder cancer (MIBC). Although ctDNA assays are not yet widely implemented in routine clinical practice, a growing body of evidence supports its utility in diagnosis, risk stratification, prognosis, therapeutic decision-making and post-treatment surveillance. This review summarizes the current and evolving roles of ctDNA in bladder cancer management, with particular emphasis on completed and ongoing clinical trials that are shaping its integration into standard care. A comprehensive literature search was conducted across PubMed, MEDLINE, Google Scholar, Embase, and Scopus for studies published between January 2005 and November 2025. The search strategy included the following terms: bladder cancer, circulating tumor DNA, liquid biopsy, circulating biomarkers. A total of 172 English-language records were initially identified. After screening for relevance to the objectives of this review, 29 studies met the inclusion criteria and were analyzed in detail. In bladder cancer, ctDNA has demonstrated prognostic value across multiple disease stages, including diagnosis, assessment of response to neoadjuvant chemotherapy, and surveillance post-cystectomy. In the adjuvant setting, ctDNA-positivity has been associated with improved outcomes when patients are subsequently treated with immune checkpoint inhibitors, as shown in the IMvigor010 trial and further explored in the ongoing IMvigor011 trial. Ongoing studies (e.g. MODERN and TOMBOLA) aim to further validate ctDNA-guided treatment strategies, and their role in prognostication and disease monitoring. Multiple studies and ongoing trials have established the utility of ctDNA in the realm of bladder cancer with indications spanning detection of recurrence, guiding use of adjuvant therapy, and prognosis evaluation. Despite this momentum, several barriers continue to limit broader clinical integration, including high testing costs, absence of standardized assay protocols, and significant inter-laboratory variability. Addressing these challenges through robust clinical trials and standardized regulatory frameworks will be essential for the widespread and reliable adoption of ctDNA in bladder cancer care.

## Plain language summary

Bladder cancer is often hard to monitor after treatment. This article explains how a simple blood test that looks for tiny pieces of tumor DNA, called circulating tumor DNA (ctDNA), can help doctors check if cancer is coming back, decide who needs extra treatment, and avoid unnecessary therapies. We review the latest research and clinical trials showing how ctDNA could make bladder cancer care more personalized, safer, and more effective.

## Introduction

Bladder cancer is the 12^th^ most common cancer worldwide, with an estimated 573,000 new cases and 212,000 related deaths reported in 2020, highlighting its considerable burden on public health.^1^ The disease is characterized by marked heterogeneity in both clinical behavior and molecular profiles. Current treatment decisions rely heavily on conventional clinicopathological features such as tumor stage, grade, nodal status, and lympho-vascular invasion. However, these parameters often fail to capture the complex underlying tumor biology or accurately predict response to treatment.^2^ The incorporation of molecular biomarkers into clinical workflows offers the potential to enhance personalized treatment strategies by enabling more refined risk stratification, dynamic disease monitoring and individualized treatment decision-making.^2^

Liquid biopsy techniques, defined as the analysis of tumor-derived material in body fluids, have gained prominence in the context of bladder cancer management.^2^ These methods are minimally invasive, easily repeatable, and allow for real-time assessment of the disease dynamics. Blood and urine represent the most accessible sources of liquid biopsy. Urine cytology remains a standard tool for both initial diagnosis and ongoing surveillance, although its limited sensitivity has prompted ongoing investigation into novel urinary biomarkers. These accessible and non-invasive characteristics facilitate broader clinical adoption.^3,4^

Parallel advances in blood-based biomarkers are increasingly being explored in the landscape of cancer diagnostics. Among liquid biopsy analytes in bladder cancer, circulating tumor cells (CTCs) and exosomes have shown potential but are limited by technical challenges, low abundance, and lack of standardization. In contrast, circulating tumor DNA (ctDNA) provides a more accessible and sensitive platform for detecting tumor-specific genomic alterations, enabling dynamic disease monitoring and improved risk stratification. These advantages have positioned ctDNA as a promising tool in the evolving landscape of precision oncology.^5^ ctDNA analysis offers a non-invasive method for detecting tumor-specific genomic alterations, offering particular value in metastatic or recurrent disease, where repeat tissue biopsies can be invasive, costly or unfeasible.^6^

This review aims to provide a comprehensive overview of ctDNA applications in bladder cancer. We focus on its evolving role in diagnosis, prognosis and treatment selection. We also highlight key completed or ongoing clinical trials that evaluated ctDNA-guided strategies and discuss future directions and challenges in integrating ctDNA testing into routine practice. This review is guided by the Scale for the Assessment of narrative review articles (SANRA).^7^

## Methods

A comprehensive literature search was undertaken on PubMed, MEDLINE, Google Scholar, Embase, and Scopus to prepare this narrative review. Peer-reviewed English-language publications from January 2005 to November 2025 were considered. The search strategy is provided in Appendix 1. Two independent reviewers screened titles and abstracts for eligibility (SSM and AA). Full-text review was conducted for potentially eligible studies. Discrepancies were resolved through discussion and, when necessary, adjudication by a senior author (JDR).

To identify ongoing and unpublished studies, a supplementary search of ClinicalTrials.gov was performed using the terms “bladder cancer,” “urothelial carcinoma,” “circulating tumor DNA,” “ctDNA,” “liquid biopsy,” and “minimal residual disease.” Recruiting and active, not-recruiting studies were reviewed, and relevant ongoing trials were incorporated into the section summarizing ongoing ctDNA-related studies. This registry search was performed separately from the primary literature search.

A total of 172 English-language records were identified and reviewed; 29 studies met the relevance criteria and were included in the final analysis. The detailed search strategy is illustrated in Figure 1. The inclusion and exclusion criteria are as follows:

**Figure 1.**
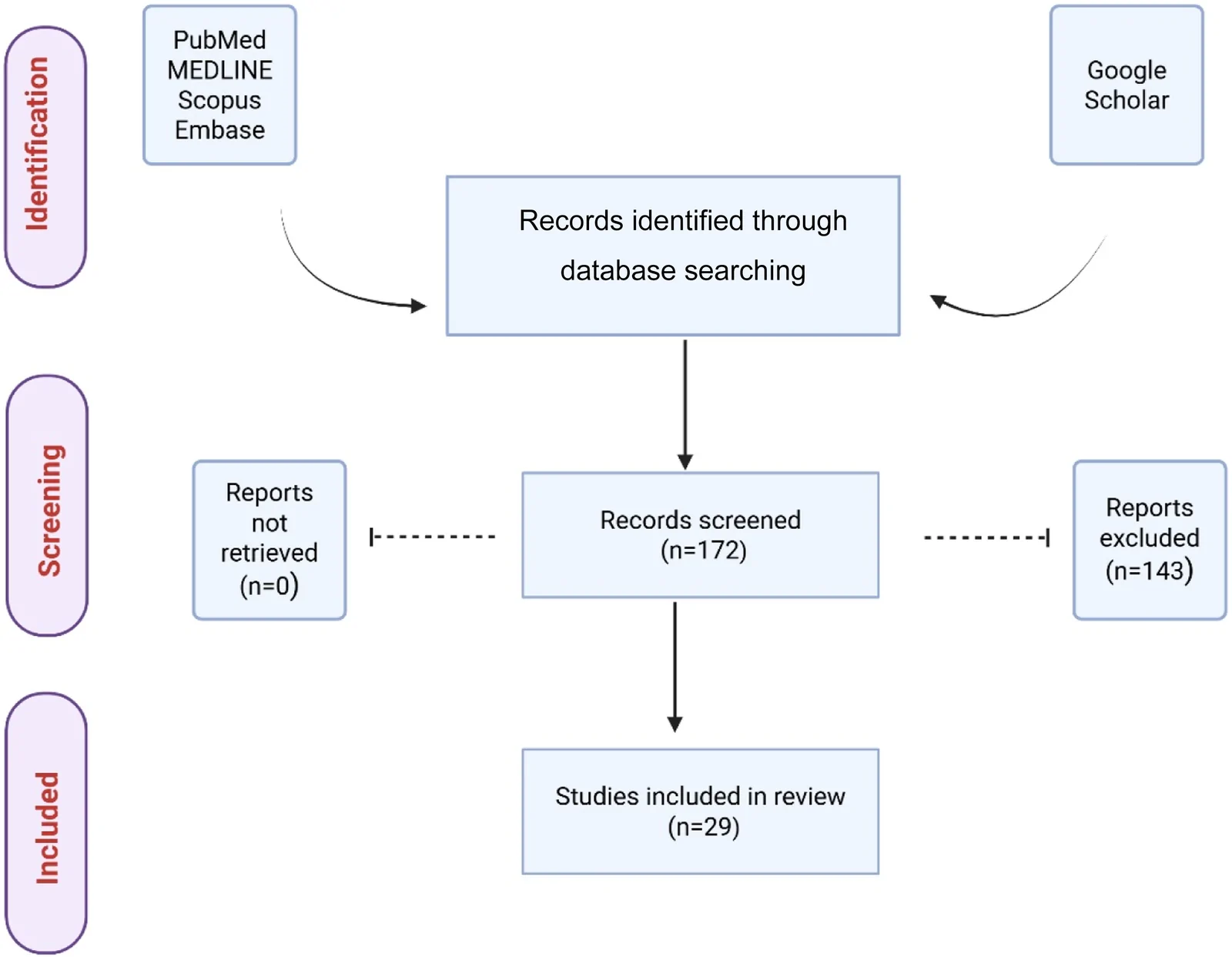
Search strategy and identification of studies for inclusion in the review.

Inclusion criteria:

1. Original studies, clinical trials, and high-quality prospective/retrospective analyses evaluating the use of circulating tumor DNA (ctDNA) in bladder cancer
2. Studies reporting diagnostic, prognostic, or therapeutic implications
3. English-language publications

Exclusion criteria:

1. Case reports, conference abstracts without full text, editorials, and review articles
2. Non-human studies
3. Studies lacking primary ctDNA-related outcomes

Because this review specifically focused on ctDNA applications in bladder cancer, studies evaluating other liquid biopsy analytes (e.g., circulating tumor cells, exosomes, urinary biomarkers) were excluded, resulting in a relatively focused pool of eligible studies.

### Definition and Characteristics

Cell-free DNA (cfDNA) refers to extracellular DNA fragments released into the circulation through apoptosis, necrosis, or active cellular secretion. In healthy individuals, most cfDNA is derived from hematopoietic cells. The interest in cfDNA and specifically ctDNA, derived from malignant cells, has surged since 2010 due to advancements in molecular detection technologies.^8^ cfDNA typically consists of short DNA fragments (about 167 base pairs in length) and has a short circulating half-life ranging from 16 minutes to 2.5 hours.^9^ ctDNA retains the tumor-specific genetic and epigenetic alterations including somatic mutations, structural rearrangement, methylation patterns and nucleosome positioning that reflect the underlying tumor biology.^10–12^ The concentration of ctDNA correlates with tumor burden, proliferative activity and treatment response.^10–12^ It is estimated that up to 3.3% of tumor DNA enters the circulation each day from 100 grams of tumor tissue (about 3 × 10^10^ tumor cells).^13,14^

Importantly, plasma cfDNA is not exclusively derived from tumors. Clonal hematopoiesis of indeterminate potential (CHIP), age-related expansions of hematopoietic stem cell clones, can introduce somatic mutations into cfDNA, potentially leading to false-positive ctDNA results.^15^ The prevalence of CHIP increases with age, occurring in roughly 1% of individuals under 50 and more than 10% of those older than 65. To minimize CHIP-related confounding, sequencing of matched white blood cell DNA is often recommended as a standard quality control measure.^16^

A major limitation of ctDNA analysis is its typically low concentration, especially in early-stage disease, which can lead to false-negative results. Detection sensitivity may also be influenced by pre-analytical factors such as sample volume, processing time, and storage conditions.^17^ Notably, ctDNA detection in bladder cancer may be limited by lower tumor DNA shedding into the bloodstream, particularly in early-stage disease. As a result, urinary tumor DNA (utDNA) may provide higher sensitivity, owing to direct tumor shedding into the urinary tract.^2,3^ Furthermore, the mutational landscape of ctDNA varies by cancer type and stage, which can influence the detection sensitivity of standard assays.^10^ Another significant challenge is the absence of standardized methodologies and consistent terminology across ctDNA platforms and laboratories. Therefore, as ctDNA testing becomes more widespread, the development of standardized guidelines will be essential to ensure analytical reliability and clinical reproducibility.^18^

Despite these challenges, ctDNA holds significant promise across the cancer care continuum. It can support early cancer detection, assess minimal residual disease, monitor treatment efficacy, identify emerging resistance mechanisms and provide insights into tumor biology across all disease stages.^19^

### Mechanisms and Detection Assays

ctDNA detection methods are broadly classified into tumor-informed and tumor-agnostic approaches, each offering distinct clinical applications. Tumor-informed assays are designed using prior knowledge of a patient’s tumor-specific mutations.^20,21^ These methods offer high sensitivity and specificity, making them particularly effective for detecting minimal residual disease (MRD) and monitoring treatment response. However, they require tumor tissue for initial profiling and typically involve longer turnaround times.^20,21^ In contrast, tumor-agnostic assays do not rely on known mutations and are more suitable for early cancer detection, diagnosis, identification of novel therapeutic targets, and metastatic disease surveillance. While these methods are standardized and faster, they are limited by lower sensitivity, reduced specificity, and inter-patient variability.^20–22^

Several technologies are employed for ctDNA detection:

• Real-time polymerase chain reaction (PCR) is widely used due to its affordability and rapid processing. This method uses fluorescent-labeled probes to detect specific nucleic acid sequences, with the fluorescence signal increasing proportionally to the amount of the amplified product.^23^
• Digital droplet PCR (ddPCR) partitions a DNA sample into thousands of nanoliter-sized droplets, enabling precise quantification of low-abundance ctDNA. This technology is used for absolute quantification, rare mutation detection, copy number variation, DNA methylation, and identification of gene rearrangements. ddPCR is valued for its ease of use, rapid workflow, and high analytical sensitivity.^24^
• BEAMing (beads, emulsion, amplification, and magnetics) integrates droplet PCR with magnetic bead-based capture to enable highly sensitive detection of ctDNA. Amplification occurs within microemulsions, and products are quantified using flow cytometry. Despite its high sensitivity, BEAMing is limited by its complex workflow and high operational costs.^25,26^
• Mass spectrometry-based assays use a two-step PCR amplification process followed by detection through mass spectrometry, offering very high sensitivity identification of specific genetic variants.^27^
• Next-generation sequencing (NGS) technologies provide comprehensive genomic, transcriptomic, and epigenetic profiling. NGS can be designed to detect known variants or applied in an untargeted manner to identify novel alterations. Whole exome sequencing (WES) focuses on protein-coding regions of the genome, while whole genome sequencing (WGS) captures both coding and noncoding regions. Both approaches typically rely on target-enrichment strategies and array-based capture techniques. However, their broad coverage is limited by high costs, longer turnaround times, and increased labor requirements.^22^

Several analytical factors influence the performance and interpretation of ctDNA assays. Variant calling approaches vary among platforms and may incorporate predefined mutation panels, tumor-informed analyses, or de novo variant detection algorithms. Sensitivity is affected by sequencing depth, background error correction methods, and the minimum threshold required to classify a variant as detectable. Variant allele frequency, defined as the proportion of sequencing reads harboring a specific mutation, is commonly used to quantify ctDNA burden and monitor dynamic changes during treatment. Modern error-corrected next-generation sequencing and digital PCR platforms can detect variants at very low allele frequencies, often below 0.1%, although analytical sensitivity varies according to assay design, sample quality, and tumor DNA shedding characteristics. Standardization of these technical parameters remains an important challenge for broader clinical implementation of ctDNA testing.

### Emerging techniques for indirect assessment of tumor transcriptional activity via ctDNA include

• DNA methylation profiling, which reflects the tumor’s epigenetic landscape.^28^
• ctDNA fragmentation analysis, providing insights into nucleosome positioning and gene expression.^28^
• Plasma-based chromatin immunoprecipitation sequencing (ChIP-Seq), which identifies histone modifications and may help detect normal cell destruction from tumor invasion and potential metastatic sites.^28^

### Clinical Applications of ctDNA

The current and potential future applications of ctDNA across the bladder cancer continuum are summarized in Figure 2.

**Figure 2.**
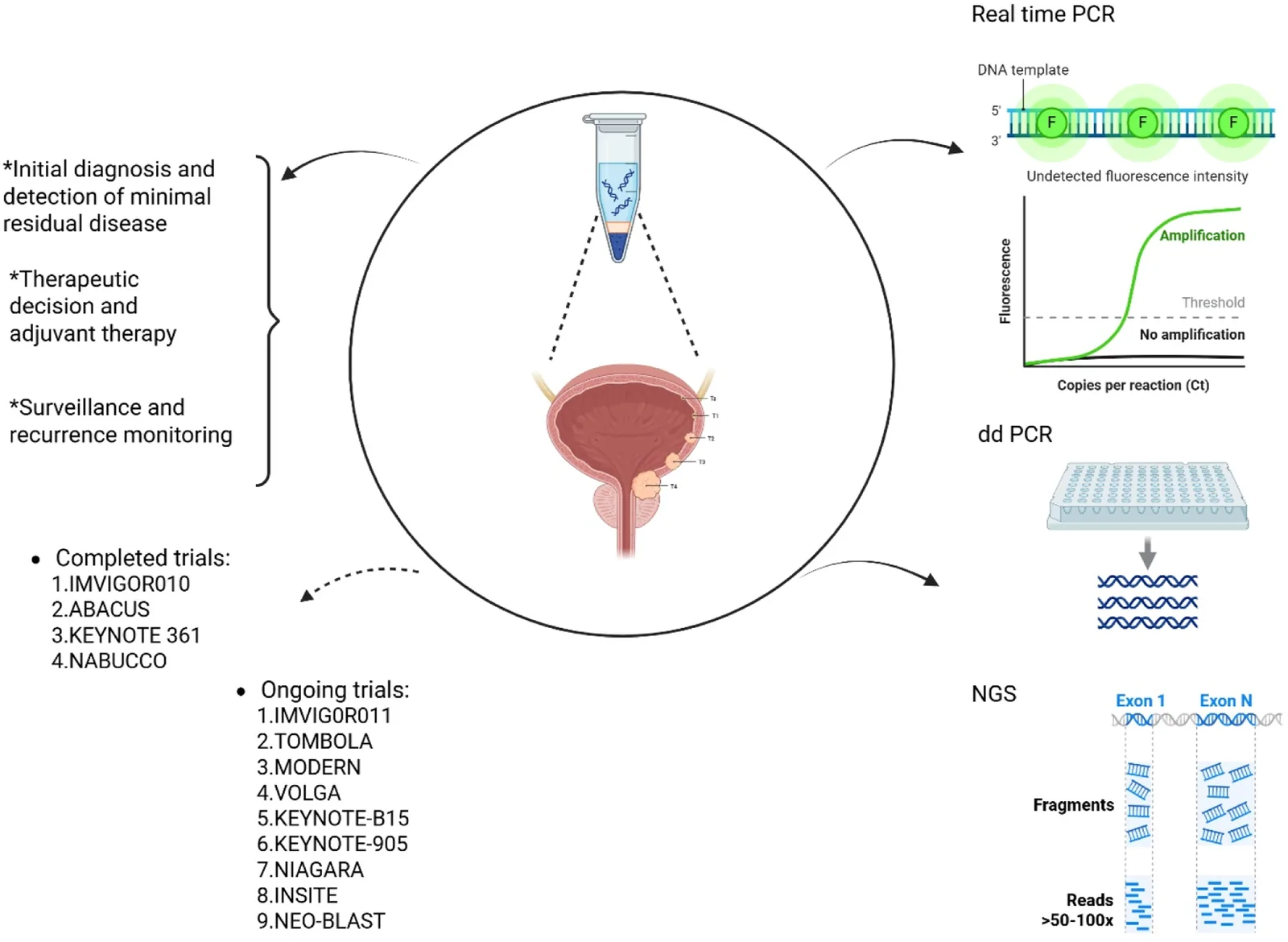
Clinical utility of ctDNA in bladder cancer with ongoing and completed trials in the realm of ctDNA in bladder cancer.

### Diagnosis and Detection of Minimal Residual Disease (MRD)

In non-muscle-invasive bladder cancer (NMIBC), emerging evidence supports the potential clinical relevance of ctDNA. In a small retrospective study (n=12), three NGS techniques were used to monitor somatic variants in tumor, plasma, and urine DNA. The study demonstrated that tumor-derived DNA was detectable in both blood and urine, with elevated levels preceding disease progression.^29^ In a larger cohort study by Zhang et al., involving 82 NMIBC patients undergoing transurethral resection and immunotherapy, ctDNA was identified in 52% of patients. The ctDNA burden was positively correlated with the initial tumor size and was a predictor of recurrence.^30^

In NMIBC, urinary tumor DNA (utDNA) — DNA detected in urine — is more informative than plasma ctDNA due to direct tumor shedding into the urinary tract. In contrast, plasma ctDNA has shown significant promise in muscle-invasive bladder cancer (MIBC).^2^ In a study of 68 patients with advanced bladder cancer, ctDNA detection at diagnosis prior to chemotherapy was significantly associated with inferior oncologic outcomes. Post-cystectomy ctDNA was also highly predictive of disease recurrence with all patients with recurrence having detectable ctDNA (100% sensitivity and 98% specificity).^31^ Notably, ctDNA detection preceded radiographic evidence of recurrence by a median of 96 days, offering a valuable lead-time window. Additionally, in patients with ctDNA positivity before treatment, ctDNA dynamics during chemotherapy were predictive of disease recurrence.^31^

The detection of ctDNA following definitive surgical intervention—ctDNA-based MRD testing—has emerged as a key prognostic tool. A retrospective study of 167 patients (852 plasma samples) showed that detectable ctDNA after surgery was associated with significantly shorter disease-free survival (DFS) (Hazard Ratio 6.93, p < 0.001). Interestingly, patients with undetectable ctDNA did not appear to benefit from immunotherapy. Detectable ctDNA at any disease stage—pre-cystectomy, during the MRD window, or in post-cystectomy surveillance—was associated with reduced DFS.^32^

Ben-David et al. further explored this concept noting that patients with undetectable ctDNA prior to cystectomy demonstrated a significantly better DFS. Such observations suggest that these patients may be optimal candidates for treatment de-escalation or surveillance-only strategies.^33^ These findings have been corroborated by additional studies.^34^

### Therapeutic Decision Making

While ctDNA has established utility in prognosis and early detection of recurrence, emerging evidence suggests that it may also contribute to therapeutic decision-making, particularly by identifying patients at higher risk of recurrence who may benefit from treatment intensification and those with favorable molecular profiles who may be candidates for treatment de-escalation. However, most currently available evidence is derived from prognostic and exploratory biomarker studies, and prospective validation of ctDNA-guided treatment strategies remains ongoing. Key completed exploratory analyses and prospective biomarker-driven trials evaluating these concepts are discussed in subsequent dedicated sections.^35–39^

### Surveillance and Recurrence Monitoring

Postoperative ctDNA level has emerged as a valuable tool for surveillance. In a prospective study of 94 patients who underwent radical cystectomy, ctDNA demonstrated a sensitivity of 100% and specificity of 91.8% in detecting recurrence.^40^ Therefore, patients with persistently undetectable ctDNA may be candidates for less intensive monitoring protocols, while early ctDNA positivity can suggest preemptive intervention with possible improvement in long-term outcomes. As discussed above, ongoing trials such as TOMBOLA and MODERN will clarify whether ctDNA negativity can be used to safely reduce the intensity of follow-up imaging and clinical visits.

### Current Evidence and Ongoing Clinical Trials

As highlighted previously, ctDNA has emerged as a promising biomarker in the management of MIBC, particularly for identifying minimal residual disease (MRD), guiding adjuvant treatment, monitoring therapeutic response and stratifying the risk of recurrence. In this section, we summarize the completed (Table 1) and ongoing (Table 2) clinical trials addressing the clinical utility of ctDNA for management of bladder cancer.

**Table 1.** Completed clinical studies evaluating the prognostic and exploratory predictive value of ctDNA in bladder cancer.

Study Name	Study period	Country	Type of Trial	Number of patients	Median follow-up	Outcome	Role of ctDNA emphasized in the study
IMvigor010^35^	2015-19	Global study involving 24 countries	Phase 3 RCT (Post-hoc analysis of role of ctDNA)	581*	46.8 months	Comparison of OS with adjuvant atezolizumab vs. observation in high-risk MIBC post-cystectomy	Prognostic biomarker for MRD and exploratory association with immunotherapy benefit
Key findings	ctDNA-positive patients had worse OS (HR 6.3); atezolizumab improved outcomes in ctDNA-positive group (HR 0.59).						
ABACUS^41^	2016-18	UK, France, Netherlands, Spain	Phase 2 single-arm (Post-hoc analysis of role of ctDNA)	95	25 months	Comparison of disease-free survival with neoadjuvant atezolizumab in MIBC who were ineligible for cisplatin	Prognostic biomarker associated with pathological response and relapse risk
Key findings	Neoadjuvant atezolizumab in MIBC is associated with clinical responses and high DFS. ctDNA negativity or clearance correlated with better pathological response and prognosis.						
KEYNOTE 361^38^	2016- 18	Global	Phase 3 (Post-hoc analysis of role of ctDNA)	263*	31.7 months	Comparison of OS with first-line pembrolizumab + chemotherapy vs chemotherapy in advanced urothelial carcinoma	Prognostic biomarker associated with OS, PFS and treatment response
Key findings	Lower baseline ctDNA associated with better OS (p < 0.001), PFS (p < 0.001), and BOR (p = 0.009) with pembrolizumab. ctDNA reduction at cycle 2 correlated with OS (p = 7.0 × 10^-5^) and BOR (p = 4.39 × 10^-5^). ctDNA changes were more predictive in pembrolizumab arm than chemotherapy. ctDNA did not independently predict OS beyond radiological assessment (P = 0.364).						
NABUCCO^42^	2018-21	Netherlands	Phase 1b (Post-hoc analysis of role of ctDNA)	24	48 months	Comparison of progression-free survival with neoadjuvant ipilimumab + nivolumab in stage III MIBC	Prognostic biomarker associated with the treatment outcomes
Key findings	The odds ratio for pCR in patients with absent ctDNA was 45 (95% CI 4.86-416.46), and hazard ratio for progression free survival was 10.4 (95% CI 2.9-37.50)						

RCT- Randomized controlled trial. DFS- Disease free survival. pCR-pathological complete remission. HR – hazard ratio. MIBC- Muscle-invasive bladder cancer. ctDNA – circulating tumor DNA. BOR – Best Overall Response. OS- Overall survival.

*ctDNA subset.

**Table 2.** Ongoing clinical trials in the realm of ctDNA and bladder cancer.

Study Name	Country	Type of Trial	Focus	ctDNA Role
TOMBOLA^39^ NCT04138628	Denmark	Phase II, nonrandomized	Early immunotherapy (atezolizumab) in ctDNA-positive MIBC after cystectomy	Biomarker for disease recurrence and early intervention
Interim results	Interim results of 153 patients, showed that only 3% of patients with ctDNA- negative had metastatic disease on CT scan.			
IMvigor011^36^ NCT04660344	Global	Phase III RCT	Atezolizumab vs. placebo in ctDNA+ MIBC patients after cystectomy	Stratification for adjuvant therapy and MRD monitoring
Interim results	Interim report showed that negative ctDNA status has excellent outcomes: 18-month OS 98%, and DFS 88%; suggesting that adjuvant therapy may potentially be omitted in this subgroup. Atezolizumab arm showed significant improvements in DFS (HR 0.64) and OS (HR 0.59).			
MODERN^37^ NCT05987241	Global	Phase II/III RCT	ctDNA-guided adjuvant therapy in MIBC	Risk stratification and treatment personalization
Results	Pending; aims to validate ctDNA as a tool for guiding adjuvant treatment decisions with nivolumab with/without relatlimab			
VOLGA^43^ NCT04960709	Global	Phase III RCT (exploratory analysis of role of ctDNA)	Neoadjuvant and adjuvant durvalumab + tremelimumab + enfortumab vedotin in MIBC cases not eligible for cisplatin	Predictive biomarker for pathological response and EFS
Interim results	Interim results of 17 patients, ctDNA clearance during neoadjuvant therapy was associated with pCR and longer EFS (HR 0.18, 95% CI 0.016-0.21)			
KEYNOTE-B15^44^ NCT04700124	Global	Phase III RCT (exploratory analysis of role of ctDNA)	Neoadjuvant pembrolizumab + chemotherapy in cisplatin-eligible MIBC	Exploratory biomarker for response and MRD
Results	Pending; evaluating ctDNA for early response prediction and relapse monitoring			
KEYNOTE-905/EV-303^45^ NCT03924895	Global	Phase III RCT (exploratory analysis of role of ctDNA)	Neoadjuvant/adjuvant pembrolizumab ± enfortumab vedotin in cisplatin-ineligible MIBC	Exploratory biomarker for treatment response and MRD
Results	Pending; assessing ctDNA for stratifying benefit from immunotherapy			
NIAGARA^46^ NCT03732677	Global	Phase III RCT (exploratory analysis of role of ctDNA)	Neoadjuvant durvalumab + chemotherapy followed by adjuvant durvalumab in MIBC	Exploratory biomarker for MRD and recurrence risk
Interim results	Interim results of 462 patients have shown that ctDNA clearance from baseline to pre-RC was 41% in durvalumab (D arm) as compared to comparator arm (31%).			
INSITE^47^ NCT06396533	USA	Observational	Diagnostic utility of combined systematic endoscopic evaluation, ctDNA and utDNA to detect residual tumor at cystectomy	MRD detection and treatment guidance
Results	Pending.			
NEO-BLAST^47^ NCT06537154	Canada	Phase II/III Randomized Study	To restage MIBC after NAC and randomly assign complete remission cases to either active surveillance or definitive surgery	Part of multi-modal restaging strategy
Results	Pending, ctDNA used to identify true complete responders eligible for active surveillance			
EORTC GUCG 2418 – STARBURST^48^	Global	Phase II single arm	Multimodal assessment of response after neoadjuvant therapy to enable future risk-adapted treatment strategies in MIBC.	Exploratory biomarker combined with imaging and other modalities to predict response; not used for prospective treatment guidance.
Results	Pending, ctDNA/utDNA part of a multimodal assessment signature.			

RCT- Randomized controlled trial. DFS- Disease free survival. EFS – event free survival. OS- overall survival. pCR-pathological complete remission. HR – hazard ratio. MIBC- Muscle-invasive bladder cancer. ctDNA – circulating tumor DNA. CT – Computed tomography. MRD – Minimal residual disease. utDNA – urinary tumor DNA. NAC – Neoadjuvant chemotherapy. RC- Radical cystectomy.

Among completed studies evaluating ctDNA in bladder cancer (Table 1), most evidence derives from exploratory, translational, or post-hoc biomarker analyses rather than prospective ctDNA-guided intervention trials. These studies have primarily established the prognostic significance of ctDNA and generated hypotheses regarding its potential predictive role in treatment selection.

IMvigor010 stands out as a pivotal Phase III randomized controlled trial evaluating adjuvant atezolizumab versus observation following radical cystectomy in patients with muscle-invasive bladder cancer. The trial did not meet its primary endpoint of disease-free survival in the overall intention-to-treat population. A post-hoc ctDNA analysis was conducted on 581 of these patients who had available ctDNA data. Results showed that ctDNA-positive patients had a survival benefit from atezolizumab (HR 0.59; 95% CI 0.42–0.83), whereas ctDNA-negative patients did not. Furthermore, survival outcomes were closely linked to the degree of circulating tumor DNA (ctDNA) reduction. Patients who achieved complete clearance (100% reduction) had a median overall survival (OS) of 60 months. Those with partial clearance (50–99% reduction) had a median survival of 34.3 months, while patients with minimal reduction (<50%) experienced the shortest survival, with a median of 19.9 months.^35^ These findings suggest that ctDNA status may help identify patients more likely to benefit from adjuvant immunotherapy. Because the ctDNA analysis was performed as a post-hoc exploratory biomarker assessment in a trial that failed its primary endpoint, the results remain hypothesis-generating and require prospective validation (currently underway in IMvigor011).^36^

The ABACUS trial, a Phase II single-arm study conducted in Europe, evaluated neoadjuvant atezolizumab in cisplatin-ineligible patients with muscle-invasive bladder cancer (MIBC). ctDNA negativity or clearance was significantly associated with improved relapse-free survival. Notably, no relapses were observed in ctDNA-negative patients at baseline or after neoadjuvant therapy. At the post-cystectomy time point, ctDNA-positive status was strongly prognostic for relapse (Hazard ratio = 78, *p* < 0.001).^41^

Similarly, post-hoc analysis of the KEYNOTE 361 trial demonstrated that lower baseline ctDNA was associated with better OS (p < 0.001), progression-free survival (PFS) (p < 0.001), and BOR (p = 0.009) with pembrolizumab. Additionally, ctDNA reduction at cycle 2 correlated with OS (p = 7.0 × 10^-5^) and BOR (p = 4.39 × 10^-5^). Notably, ctDNA changes were more predictive in the pembrolizumab arm than in the chemotherapy, and ctDNA did not independently predict OS beyond radiological assessment (P = 0.364).^38^ As with IMvigor010 and ABACUS, ctDNA was evaluated as an exploratory biomarker and was not incorporated into treatment decision-making.

Likewise, the NABUCCO trial, a Phase Ib single-arm study, evaluated neoadjuvant ipilimumab plus nivolumab in patients with stage III muscle-invasive bladder cancer (MIBC). On exploratory analysis, the absence of ctDNA was strongly associated with pathological complete response (pCR) (odds ratio [OR]= 45, p < 0.01), and with improved relapse-free survival (RFS) (hazard ratio [HR]= 10.4, *p*<0.001). These findings demonstrate a strong association between ctDNA status and treatment outcomes. However, because ctDNA was evaluated retrospectively and did not guide therapy, the results should be interpreted primarily as evidence of prognostic rather than predictive utility.^42^

Collectively, these exploratory and post-hoc analyses establish the strong prognostic value of ctDNA but do not constitute prospective evidence that ctDNA can be used to guide treatment allocation. Conclusions regarding predictive utility therefore remain hypothesis-generating pending results from dedicated biomarker-driven trials.

Prospective ctDNA-guided intervention trials (Table 2):

Several prospective clinical trials are currently investigating the role of ctDNA as a biomarker to guide perioperative and adjuvant treatment strategies in MIBC. These studies collectively aim to determine whether ctDNA can be used to identify patients at high risk of recurrence who may benefit from treatment escalation, as well as those with favorable molecular responses who could potentially avoid overtreatment through de-escalation strategies.^42–46^

Biomarker-driven treatment trials

• TOMBOLA (Denmark, Phase II; NCT04138628): Investigates the benefits of early immunotherapy with atezolizumab only in ctDNA-positive patients after cystectomy. Preliminary data (n = 153) showed that only 3% of ctDNA-negative patients had radiologic evidence of metastasis, supporting the hypothesis that adjuvant immunotherapy may be preferentially considered in ctDNA-positive patients. Interim results are promising; final results are awaited. TOMBOLA represents one of the first prospective studies directly evaluating a ctDNA-guided treatment strategy.^39^
• IMvigor011 (Global, Phase III; NCT04660344): Prospectively evaluates adjuvant atezolizumab versus placebo in patients with high-risk MIBC who remain ctDNA-positive following radical cystectomy. An interim surveillance analysis presented at the European Society for Medical Oncology (ESMO) 2025 Congress focused on 144 ctDNA-negative patients who completed serial ctDNA surveillance and radiographic evaluation. Those with persistently negative ctDNA status demonstrated excellent outcomes: 18-month OS was 98%, and DFS was 88%. These findings suggest that persistently ctDNA-negative patients may represent a lower-risk subgroup for whom treatment de-escalation strategies warrant further prospective evaluation. On the other hand, ctDNA-positive patients treated with atezolizumab showed improved DFS (HR 0.64) and OS (HR 0.59).^36^
• MODERN (Global, Phase II/III; NCT05987241): Aims to validate ctDNA-guided adjuvant therapy using nivolumab ± relatlimab. Patients are stratified after cystectomy into ctDNA-positive and ctDNA-negative arms. ctDNA-positive patients are randomized to nivolumab alone or nivolumab + relatlimab; ctDNA-negative patients are randomized to nivolumab or surveillance. The primary outcome of interest is overall survival.^37^
• INSITE (USA, Observational; NCT06396533): Evaluates the diagnostic utility of combined systematic endoscopic evaluation, ctDNA and utDNA to detect residual tumor at cystectomy (MRD detection and treatment guidance). Results pending.^47^
• NEO-BLAST (Canada, Phase II/III Feasibility and Randomized Study; NCT06537154): Restages MIBC after neoadjuvant chemotherapy and randomly assigns complete-remission cases to either active surveillance or definitive surgery. ctDNA is used as part of a multi-modal restaging strategy to identify true complete responders eligible for active surveillance.^47^

Exploratory prognostic/predictive analyses within ongoing interventional trials

• VOLGA (NCT04960709): A global phase 3, multicenter, randomized trial of durvalumab in combination with tremelimumab and enfortumab vedotin or durvalumab plus enfortumab vedotin for perioperative treatment in cisplatin-ineligible (or refusing) patients undergoing radical cystectomy for MIBC. Early data link ctDNA clearance to improved pCR and event-free survival (EFS).^43^
• KEYNOTE-B15 (NCT04700124): A phase 3, randomized, open-label study evaluating perioperative enfortumab vedotin plus pembrolizumab versus neoadjuvant gemcitabine and cisplatin in cisplatin-eligible participants with MIBC. Sub-group analysis stratified by ctDNA status is planned.^44^
• KEYNOTE-905 (NCT03924895): A randomized phase 3 study evaluating cystectomy with perioperative pembrolizumab ± enfortumab vedotin versus cystectomy alone in cisplatin-ineligible or declining participants. Sub-group analysis stratified by ctDNA status is planned.^45^
• NIAGARA (NCT03732677): Examines ctDNA dynamics during neoadjuvant durvalumab plus chemotherapy (gemcitabine and cisplatin) for MIBC planned for cystectomy. Interim results of 462 patients showed ctDNA clearance from baseline to pre-RC of 41% in the durvalumab arm versus 31% in the comparator arm.^46^

Prospective biomarker assessment of response (non-interventional for treatment allocation)

• EORTC GUCG 2418 – STARBURST (Global, Phase II single-arm): Multimodal assessment of response after neoadjuvant therapy to enable future risk-adapted treatment strategies in MIBC. ctDNA/utDNA form part of an exploratory multimodal assessment signature combined with imaging; not used for prospective treatment guidance. Results pending.^48^

These trials highlight the transformative potential of ctDNA in personalizing bladder cancer treatment, namely risk-adapted management, treatment de-escalation, and early intervention. As evidence continues to mature, ctDNA is poised to become an integral component of clinical decision-making, enabling earlier intervention, improved risk stratification, and more individualized therapeutic strategies.

### Future Directions

Particularly in MIBC, ctDNA has demonstrated utility at multiple stages, including early diagnosis, prediction of response to neoadjuvant therapy, disease monitoring, and prediction of postoperative recurrence (Figure 2).^48^ Notably, ctDNA can predict postoperative recurrence risk up to three months before radiologic imaging, offering a critical window for early therapeutic intervention.^32^ This makes it a powerful tool for implementing risk-adapted adjuvant therapy and dynamic surveillance protocols.

In the adjuvant setting, ctDNA shows promise as a prognostic biomarker and may ultimately serve as a predictive biomarker for treatment benefit, although prospective validation remains ongoing. (IMvigor011).^36^

Meanwhile, in NMIBC, urinary tumor DNA (utDNA) is emerging as a sensitive biomarker for recurrence monitoring, potentially expanding the use of liquid biopsy in early-stage disease.^2,49^

Compared to traditional tissue biopsies, ctDNA testing offers several advantages: it is non-invasive, allows for dynamic disease monitoring, and provides high sensitivity for detecting tumor-specific genetic alterations. These features introduce ctDNA as a valuable tool for personalized, precision-based treatment strategies. However, sensitivity remains suboptimal in NMIBC where ctDNA levels are often below the detection thresholds.^50,51^

To overcome this limitation, several technological and methodological innovations are under investigation. High-throughput sequencing (HTS) platforms—such as error-corrected sequencing and digital droplet PCR (ddPCR) can enhance detection of ultra-low levels of ctDNA by minimizing background noise and enhancing rare mutation detection.^52^

Moreover, integrating ctDNA analysis with complementary biomarkers—such as protein markers or epigenetic features like DNA methylation—may further enhance early detection. Personalized ctDNA assays targeting patient-specific mutations or tumor-derived variants are also being developed to improve sensitivity by focusing on known genomic alterations.^53^ The application of machine learning algorithms to interpret complex genomic data adds another promising dimension, potentially enabling earlier and more accurate detection of bladder cancer.^54^

Several barriers must be addressed before ctDNA testing can be fully integrated into clinical practice. These include high testing costs, lack of standardized protocols for sample collection and analysis, and regulatory barriers. Inter-laboratory variability in ctDNA extraction, quantification, and interpretation can lead to inconsistent results, undermining clinical reliability. These challenges must be addressed through large-scale multicenter clinical trials, standardization initiatives, and strategies to reduce costs.^25,55,56^ Emerging technologies beyond sequencing, including cfDNA fragmentomics and whole-genome methylation analysis, are increasingly being explored to improve detection sensitivity. In parallel, the concept of “molecular response,” defined by ctDNA clearance or dynamic change during treatment, is gaining traction as a potential surrogate endpoint in clinical trials.^57,58^

## Conclusions

In conclusion, ctDNA holds substantial promise as a non-invasive, dynamic biomarker for personalized bladder cancer management. While its role is well-supported in advanced and high-risk MIBC, expanding its application in early-stage disease and standardizing implementation protocols are key next steps. With continued technological advancement, regulatory alignment, and prospective validation, ctDNA is poised to become a cornerstone of personalized care in detection, treatment and monitoring of bladder cancer.

## Considerations for future clinical integration of ctDNA

The following considerations are intended to highlight potential future applications of ctDNA in bladder cancer management based on currently available evidence. Although numerous studies have demonstrated the prognostic significance of ctDNA and several prospective trials are underway, routine incorporation of ctDNA into clinical decision-making remains investigational and requires further validation through prospective randomized studies.

ctDNA shows promise for risk stratification and may complement established clinicopathologic factors in future risk-adapted management strategies Emerging evidence suggests that ctDNA may help stratify recurrence risk after cystectomy, and ongoing prospective trials are evaluating whether this information can be used to guide adjuvant therapy. ctDNA has demonstrated potential for early detection of molecular recurrence; however, its role in routine surveillance requires validation in prospective randomized studies.

Follow ongoing studies such as MODERN and TOMBOLA, which are refining ctDNA-guided treatment algorithms and may influence future clinical practice.

## Data Availability

This is a narrative review, and the search strategy is provided in Appendix 1.*

## Appendix

### Appendix A

(“circulating tumor DNA” OR “circulating tumour DNA” OR ctDNA OR “cell-free DNA” OR cfDNA OR “liquid biopsy”)

AND

(“urothelial carcinoma” OR “urothelial cancer” OR “bladder cancer” OR “transitional cell carcinoma”).
